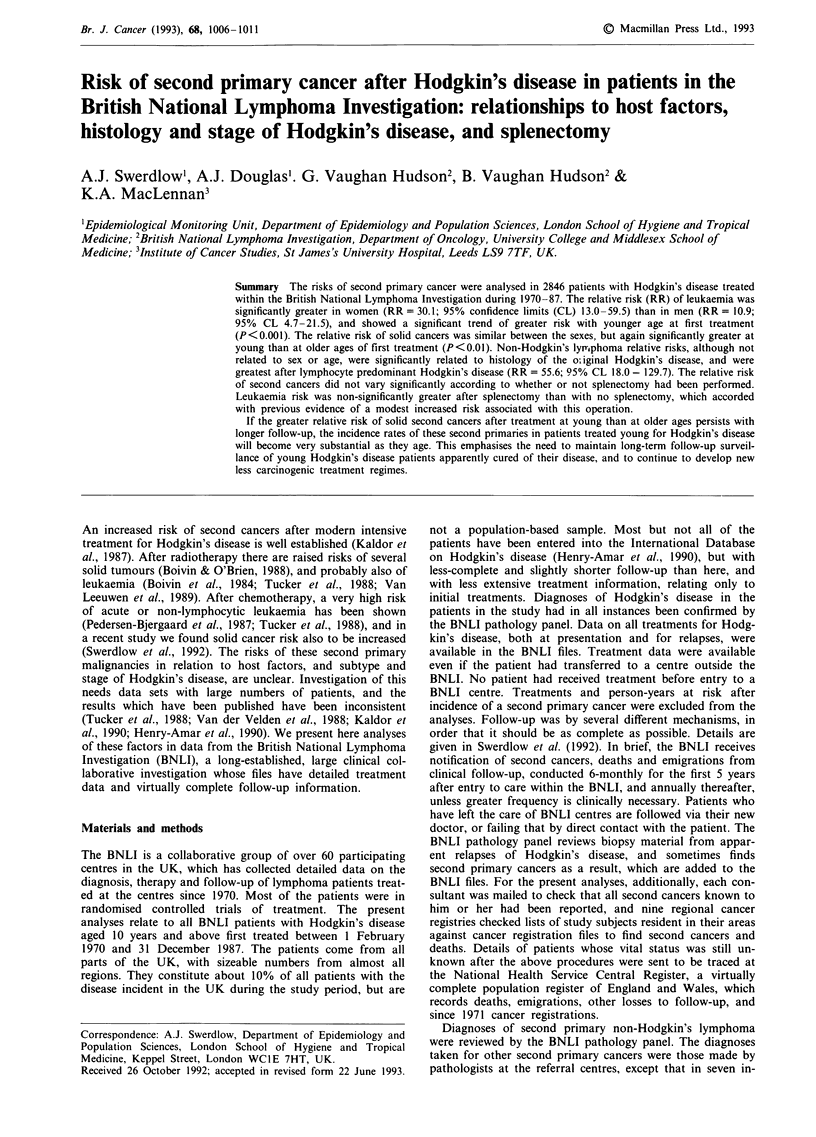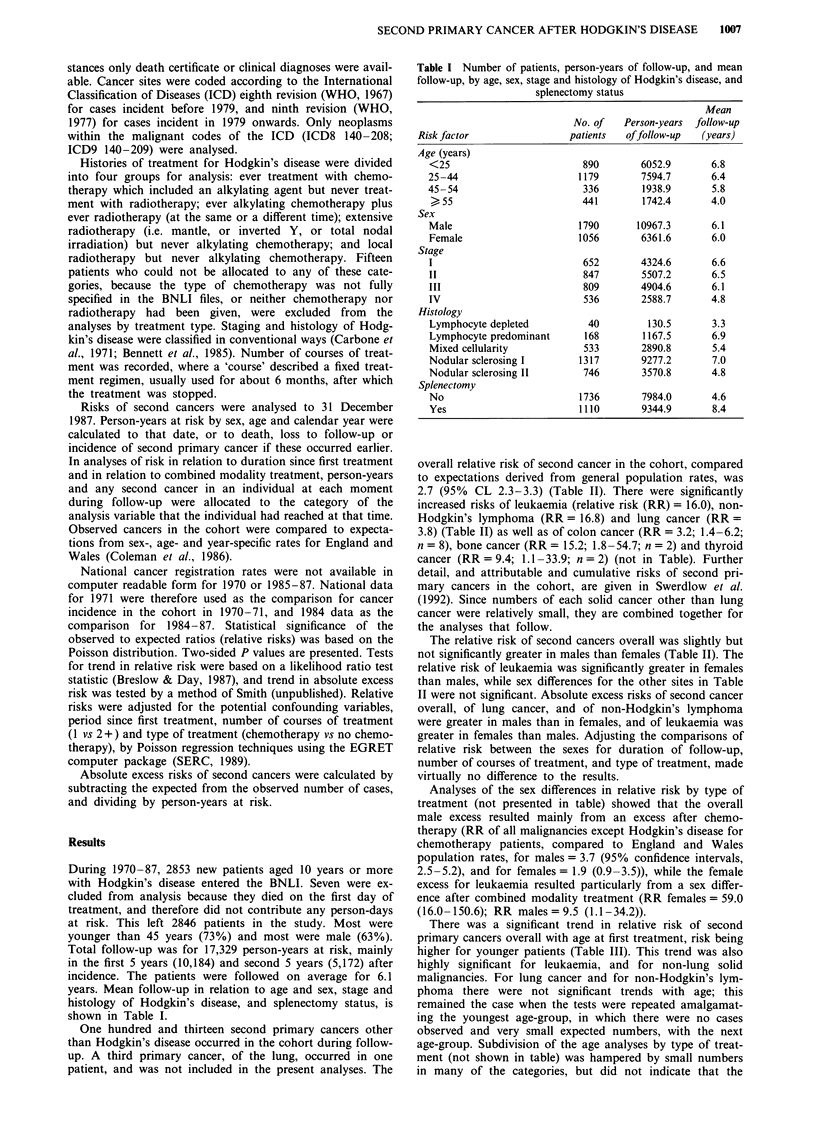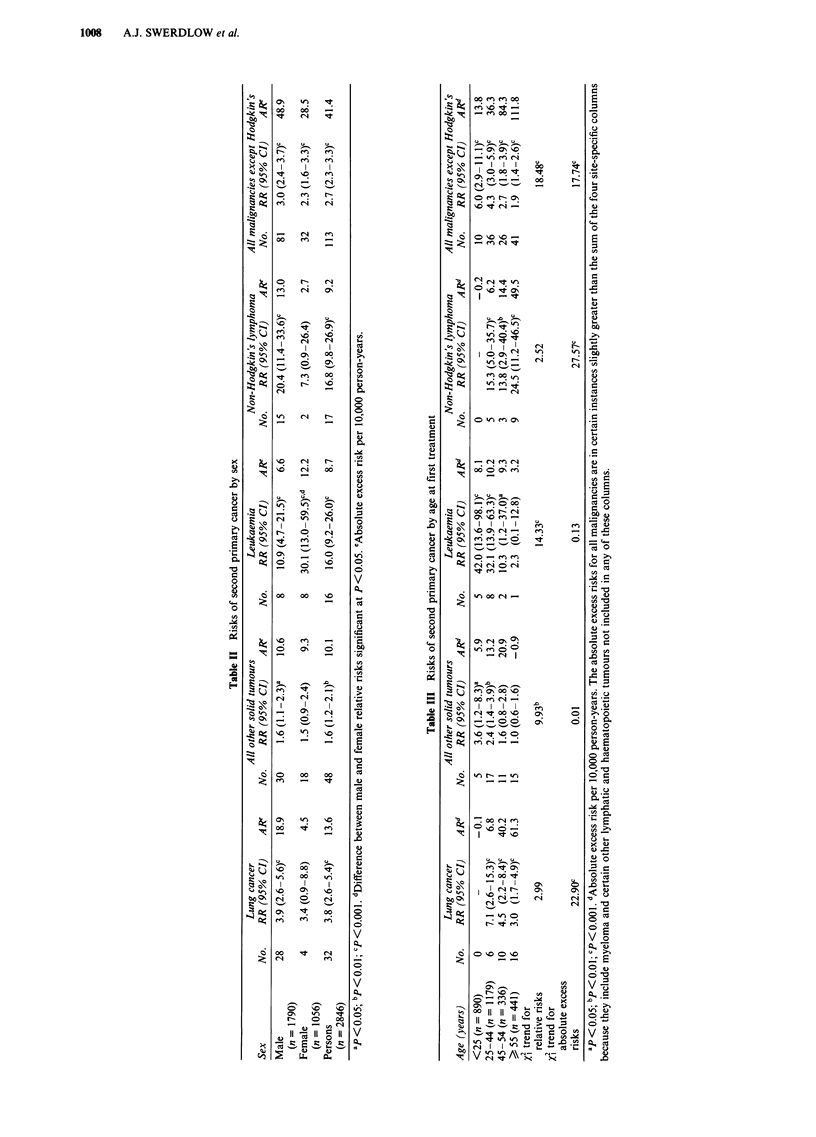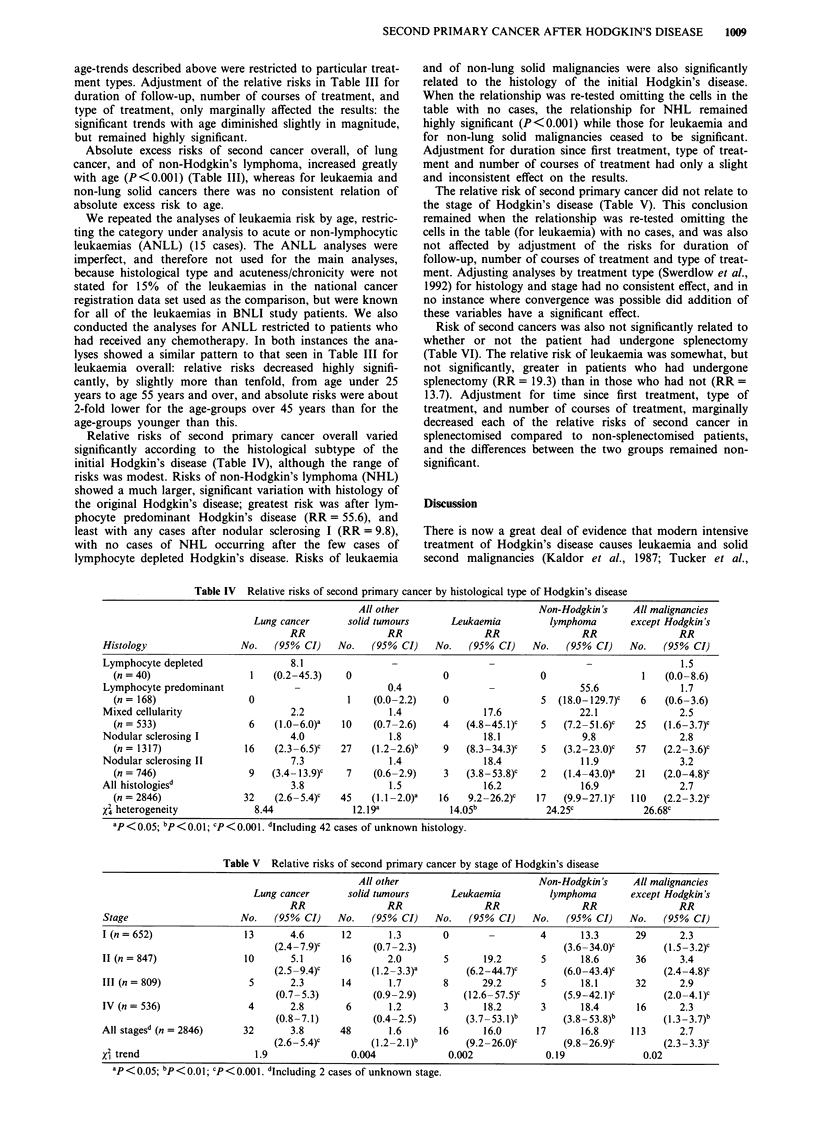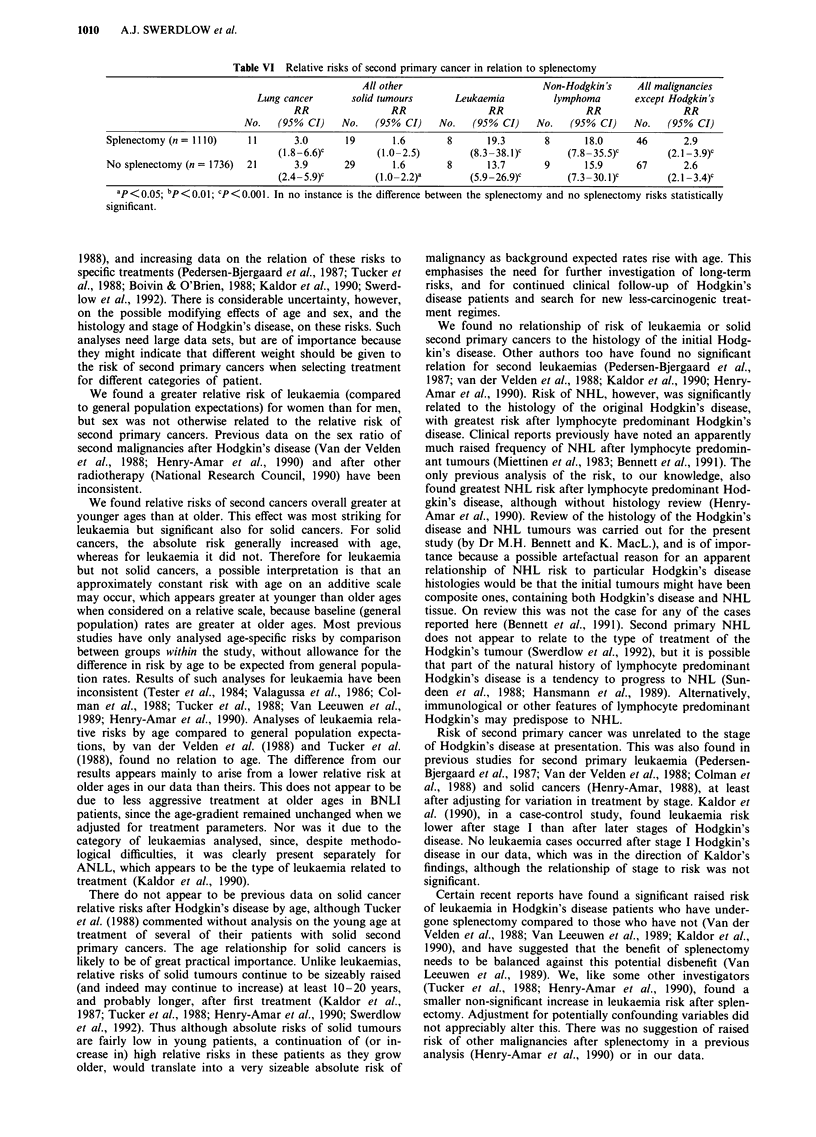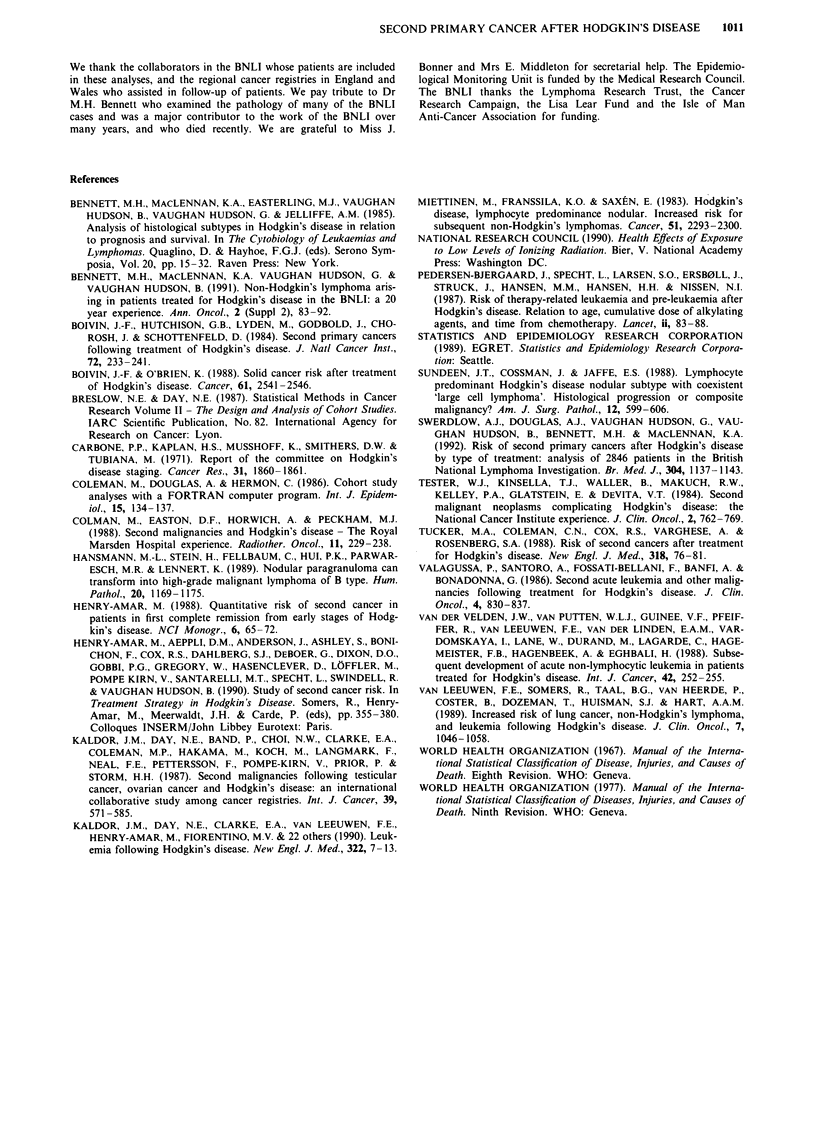# Risk of second primary cancer after Hodgkin's disease in patients in the British National Lymphoma Investigation: relationships to host factors, histology and stage of Hodgkin's disease, and splenectomy.

**DOI:** 10.1038/bjc.1993.470

**Published:** 1993-11

**Authors:** A. J. Swerdlow, A. J. Douglas, G. Vaughan Hudson, B. Vaughan Hudson, K. A. MacLennan

**Affiliations:** Department of Epidemiology and Population Sciences, London School of Hygiene and Tropical Medicine, UK.

## Abstract

The risks of second primary cancer were analysed in 2846 patients with Hodgkin's disease treated within the British National Lymphoma Investigation during 1970-87. The relative risk (RR) of leukaemia was significantly greater in women (RR = 30.1; 95% confidence limits (CL) 13.0-59.5) than in men (RR = 10.9; 95% CL 4.7-21.5), and showed a significant trend of greater risk with younger age at first treatment (P < 0.001). The relative risk of solid cancers was similar between the sexes, but again significantly greater at young than at older ages of first treatment (P < 0.01). Non-Hodgkin's lymphoma relative risks, although not related to sex or age, were significantly related to histology of the original Hodgkin's disease, and were greatest after lymphocyte predominant Hodgkin's disease (RR = 55.6; 95% CL 18.0-129.7). The relative risk of second cancers did not vary significantly according to whether or not splenectomy had been performed. Leukaemia risk was non-significantly greater after splenectomy than with no splenectomy, which accorded with previous evidence of a modest increased risk associated with this operation. If the greater relative risk of solid second cancers after treatment at young than at older ages persists with longer follow-up, the incidence rates of these second primaries in patients treated young for Hodgkin's disease will become very substantial as they age. This emphasises the need to maintain long-term follow-up surveillance of young Hodgkin's disease patients apparently cured of their disease, and to continue to develop new less carcinogenic treatment regimens.


Br. J. Cancer (1993), 68, 1006-1011 © Macmillan Press Ltd., 1993

### Risk of second primary cancer after Hodgkin's disease in patients in the British National Lymphoma Investigation: relationships to host factors, histology and stage of Hodgkin's disease, and splenectomy

A.J. Swerdlow[1], A.J. Douglas[1]. G. Vaughan Hudson[2], B. Vaughan Hudson[2] & K.A. MacLennan[3]

[1]*Epidemiological Monitoring Unit, Department of Epidemiology and Population Sciences, London School of Hygiene and Tropical Medicine;* [2]*British National Lymphoma Investigation, Department of Oncology, University College and Middlesex School of Medicine;* [3]*Institute of Cancer Studies, St James's University Hospital, Leeds LS9 7TF, UK.*

**Summary** The risks of second primary cancer were analysed in 2846 patients with Hodgkin's disease treated within the British National Lymphoma Investigation during 1970–87. The relative risk (RR) of leukaemia was significantly greater in women (RR = 30.1; 95% confidence limits (CL) 13.0–59.5) than in men (RR = 10.9; 95% CL 4.7–21.5), and showed a significant trend of greater risk with younger age at first treatment ($P < 0.001$). The relative risk of solid cancers was similar between the sexes, but again significantly greater at young than at older ages of first treatment ($P < 0.01$). Non-Hodgkin's lymphoma relative risks, although not related to sex or age, were significantly related to histology of the original Hodgkin's disease, and were greatest after lymphocyte predominant Hodgkin's disease (RR = 55.6; 95% CL 18.0 – 129.7). The relative risk of second cancers did not vary significantly according to whether or not splenectomy had been performed. Leukaemia risk was non-significantly greater after splenectomy than with no splenectomy, which accorded with previous evidence of a modest increased risk associated with this operation.

If the greater relative risk of solid second cancers after treatment at young than at older ages persists with longer follow-up, the incidence rates of these second primaries in patients treated young for Hodgkin's disease will become very substantial as they age. This emphasises the need to maintain long-term follow-up surveillance of young Hodgkin's disease patients apparently cured of their disease, and to continue to develop new less carcinogenic treatment regimes.

An increased risk of second cancers after modern intensive treatment for Hodgkin's disease is well established (Kaldor *et al.*, 1987). After radiotherapy there are raised risks of several solid tumours (Boivin & O'Brien, 1988), and probably also of leukaemia (Boivin *et al.*, 1984; Tucker *et al.*, 1988; Van Leeuwen *et al.*, 1989). After chemotherapy, a very high risk of acute or non-lymphocytic leukaemia has been shown (Pedersen-Bjergaard *et al.*, 1987; Tucker *et al.*, 1988), and in a recent study we found solid cancer risk also to be increased (Swerdlow *et al.*, 1992). The risks of these second primary malignancies in relation to host factors, and subtype and stage of Hodgkin's disease, are unclear. Investigation of this needs data sets with large numbers of patients, and the results which have been published have been inconsistent (Tucker *et al.*, 1988; Van der Velden *et al.*, 1988; Kaldor *et al.*, 1990; Henry-Amar *et al.*, 1990). We present here analyses of these factors in data from the British National Lymphoma Investigation (BNLI), a long-established, large clinical collaborative investigation whose files have detailed treatment data and virtually complete follow-up information.

### Materials and methods

The BNLI is a collaborative group of over 60 participating centres in the UK, which has collected detailed data on the diagnosis, therapy and follow-up of lymphoma patients treated at the centres since 1970. Most of the patients were in randomised controlled trials of treatment. The present analyses relate to all BNLI patients with Hodgkin's disease aged 10 years and above first treated between 1 February 1970 and 31 December 1987. The patients come from all parts of the UK, with sizeable numbers from almost all regions. They constitute about 10% of all patients with the disease incident in the UK during the study period, but are not a population-based sample. Most but not all of the patients have been entered into the International Database on Hodgkin's disease (Henry-Amar *et al.*, 1990), but with less-complete and slightly shorter follow-up than here, and with less extensive treatment information, relating only to initial treatments. Diagnoses of Hodgkin's disease in the patients in the study had in all instances been confirmed by the BNLI pathology panel. Data on all treatments for Hodgkin's disease, both at presentation and for relapses, were available in the BNLI files. Treatment data were available even if the patient had transferred to a centre outside the BNLI. No patient had received treatment before entry to a BNLI centre. Treatments and person-years at risk after incidence of a second primary cancer were excluded from the analyses. Follow-up was by several different mechanisms, in order that it should be as complete as possible. Details are given in Swerdlow *et al.* (1992). In brief, the BNLI receives notification of second cancers, deaths and emigrations from clinical follow-up, conducted 6-monthly for the first 5 years after entry to care within the BNLI, and annually thereafter, unless greater frequency is clinically necessary. Patients who have left the care of BNLI centres are followed via their new doctor, or failing that by direct contact with the patient. The BNLI pathology panel reviews biopsy material from apparent relapses of Hodgkin's disease, and sometimes finds second primary cancers as a result, which are added to the BNLI files. For the present analyses, additionally, each consultant was mailed to check that all second cancers known to him or her had been reported, and nine regional cancer registries checked lists of study subjects resident in their areas against cancer registration files to find second cancers and deaths. Details of patients whose vital status was still unknown after the above procedures were sent to be traced at the National Health Service Central Register, a virtually complete population register of England and Wales, which records deaths, emigrations, other losses to follow-up, and since 1971 cancer registrations.

Diagnoses of second primary non-Hodgkin's lymphoma were reviewed by the BNLI pathology panel. The diagnoses taken for other second primary cancers were those made by pathologists at the referral centres, except that in seven in-

Correspondence: A.J. Swerdlow, Department of Epidemiology and Population Sciences, London School of Hygiene and Tropical Medicine, Keppel Street, London WC1E 7HT, UK.
Received 26 October 1992; accepted in revised form 22 June 1993.

stances only death certificate or clinical diagnoses were available. Cancer sites were coded according to the International Classification of Diseases (ICD) eighth revision (WHO, 1967) for cases incident before 1979, and ninth revision (WHO, 1977) for cases incident in 1979 onwards. Only neoplasms within the malignant codes of the ICD (ICD8 140–208; ICD9 140–209) were analysed.

Histories of treatment for Hodgkin's disease were divided into four groups for analysis: ever treatment with chemotherapy which included an alkylating agent but never treatment with radiotherapy; ever alkylating chemotherapy plus ever radiotherapy (at the same or a different time); extensive radiotherapy (i.e. mantle, or inverted Y, or total nodal irradiation) but never alkylating chemotherapy; and local radiotherapy but never alkylating chemotherapy. Fifteen patients who could not be allocated to any of these categories, because the type of chemotherapy was not fully specified in the BNLI files, or neither chemotherapy nor radiotherapy had been given, were excluded from the analyses by treatment type. Staging and histology of Hodgkin's disease were classified in conventional ways (Carbone *et al.*, 1971; Bennett *et al.*, 1985). Number of courses of treatment was recorded, where a 'course' described a fixed treatment regimen, usually used for about 6 months, after which the treatment was stopped.

Risks of second cancers were analysed to 31 December 1987. Person-years at risk by sex, age and calendar year were calculated to that date, or to death, loss to follow-up or incidence of second primary cancer if these occurred earlier. In analyses of risk in relation to duration since first treatment and in relation to combined modality treatment, person-years and any second cancer in an individual at each moment during follow-up were allocated to the category of the analysis variable that the individual had reached at that time. Observed cancers in the cohort were compared to expectations from sex-, age- and year-specific rates for England and Wales (Coleman *et al.*, 1986).

National cancer registration rates were not available in computer readable form for 1970 or 1985–87. National data for 1971 were therefore used as the comparison for cancer incidence in the cohort in 1970–71, and 1984 data as the comparison for 1984–87. Statistical significance of the observed to expected ratios (relative risks) was based on the Poisson distribution. Two-sided *P* values are presented. Tests for trend in relative risk were based on a likelihood ratio test statistic (Breslow & Day, 1987), and trend in absolute excess risk was tested by a method of Smith (unpublished). Relative risks were adjusted for the potential confounding variables, period since first treatment, number of courses of treatment (1 *vs* 2+) and type of treatment (chemotherapy *vs* no chemotherapy), by Poisson regression techniques using the EGRET computer package (SERC, 1989).

Absolute excess risks of second cancers were calculated by subtracting the expected from the observed number of cases, and dividing by person-years at risk.

### Results

During 1970–87, 2853 new patients aged 10 years or more with Hodgkin's disease entered the BNLI. Seven were excluded from analysis because they died on the first day of treatment, and therefore did not contribute any person-days at risk. This left 2846 patients in the study. Most were younger than 45 years (73%) and most were male (63%). Total follow-up was for 17,329 person-years at risk, mainly in the first 5 years (10,184) and second 5 years (5,172) after incidence. The patients were followed on average for 6.1 years. Mean follow-up in relation to age and sex, stage and histology of Hodgkin's disease, and splenectomy status, is shown in Table I.

One hundred and thirteen second primary cancers other than Hodgkin's disease occurred in the cohort during follow-up. A third primary cancer, of the lung, occurred in one patient, and was not included in the present analyses. The overall relative risk of second cancer in the cohort, compared to expectations derived from general population rates, was 2.7 (95% CL 2.3–3.3) (Table II). There were significantly increased risks of leukaemia (relative risk (RR) = 16.0), non-Hodgkin's lymphoma (RR = 16.8) and lung cancer (RR = 3.8) (Table II) as well as of colon cancer (RR = 3.2; 1.4–6.2; *n* = 8), bone cancer (RR = 15.2; 1.8–54.7; *n* = 2) and thyroid cancer (RR = 9.4; 1.1–33.9; *n* = 2) (not in Table). Further detail, and attributable and cumulative risks of second primary cancers in the cohort, are given in Swerdlow *et al.* (1992). Since numbers of each solid cancer other than lung cancer were relatively small, they are combined together for the analyses that follow.

**Table I** Number of patients, person-years of follow-up, and mean follow-up, by age, sex, stage and histology of Hodgkin's disease, and splenectomy status

| *Risk factor* | *No. of patients* | *Person-years of follow-up* | *Mean follow-up (years)* |
|---|---|---|---|
| *Age* (years) | | | |
| <25 | 890 | 6052.9 | 6.8 |
| 25–44 | 1179 | 7594.7 | 6.4 |
| 45–54 | 336 | 1938.9 | 5.8 |
| ⩾55 | 441 | 1742.4 | 4.0 |
| *Sex* | | | |
| Male | 1790 | 10967.3 | 6.1 |
| Female | 1056 | 6361.6 | 6.0 |
| *Stage* | | | |
| I | 652 | 4324.6 | 6.6 |
| II | 847 | 5507.2 | 6.5 |
| III | 809 | 4904.6 | 6.1 |
| IV | 536 | 2588.7 | 4.8 |
| *Histology* | | | |
| Lymphocyte depleted | 40 | 130.5 | 3.3 |
| Lymphocyte predominant | 168 | 1167.5 | 6.9 |
| Mixed cellularity | 533 | 2890.8 | 5.4 |
| Nodular sclerosing I | 1317 | 9277.2 | 7.0 |
| Nodular sclerosing II | 746 | 3570.8 | 4.8 |
| *Splenectomy* | | | |
| No | 1736 | 7984.0 | 4.6 |
| Yes | 1110 | 9344.9 | 8.4 |

The relative risk of second cancers overall was slightly but not significantly greater in males than females (Table II). The relative risk of leukaemia was significantly greater in females than males, while sex differences for the other sites in Table II were not significant. Absolute excess risks of second cancer overall, of lung cancer, and of non-Hodgkin's lymphoma were greater in males than in females, and of leukaemia was greater in females than males. Adjusting the comparisons of relative risk between the sexes for duration of follow-up, number of courses of treatment, and type of treatment, made virtually no difference to the results.

Analyses of the sex differences in relative risk by type of treatment (not presented in table) showed that the overall male excess resulted mainly from an excess after chemotherapy (RR of all malignancies except Hodgkin's disease for chemotherapy patients, compared to England and Wales population rates, for males = 3.7 (95% confidence intervals, 2.5–5.2), and for females = 1.9 (0.9–3.5)), while the female excess for leukaemia resulted particularly from a sex difference after combined modality treatment (RR females = 59.0 (16.0–150.6); RR males = 9.5 (1.1–34.2)).

There was a significant trend in relative risk of second primary cancers overall with age at first treatment, risk being higher for younger patients (Table III). This trend was also highly significant for leukaemia, and for non-lung solid malignancies. For lung cancer and for non-Hodgkin's lymphoma there were not significant trends with age; this remained the case when the tests were repeated amalgamating the youngest age-group, in which there were no cases observed and very small expected numbers, with the next age-group. Subdivision of the age analyses by type of treatment (not shown in table) was hampered by small numbers in many of the categories, but did not indicate that the

**Table II** Risks of second primary cancer by sex

| Sex | Lung cancer No. | Lung cancer RR (95% CI) | Lung cancer AR[e] | All other solid tumours No. | All other solid tumours RR (95% CI) | All other solid tumours AR[e] | Leukaemia No. | Leukaemia RR (95% CI) | Leukaemia AR[e] | Non-Hodgkin's lymphoma No. | Non-Hodgkin's lymphoma RR (95% CI) | Non-Hodgkin's lymphoma AR[e] | All malignancies except Hodgkin's No. | All malignancies except Hodgkin's RR (95% CI) | All malignancies except Hodgkin's AR[e] |
|---|---|---|---|---|---|---|---|---|---|---|---|---|---|---|---|
| Male ($n$ = 1790) | 28 | 3.9 (2.6–5.6)[c] | 18.9 | 30 | 1.6 (1.1–2.3)[a] | 10.6 | 8 | 10.9 (4.7–21.5)[c] | 6.6 | 15 | 20.4 (11.4–33.6)[c] | 13.0 | 81 | 3.0 (2.4–3.7)[c] | 48.9 |
| Female ($n$ = 1056) | 4 | 3.4 (0.9–8.8) | 4.5 | 18 | 1.5 (0.9–2.4) | 9.3 | 8 | 30.1 (13.0–59.5)[c,d] | 12.2 | 2 | 7.3 (0.9–26.4) | 2.7 | 32 | 2.3 (1.6–3.3)[c] | 28.5 |
| Persons ($n$ = 2846) | 32 | 3.8 (2.6–5.4)[c] | 13.6 | 48 | 1.6 (1.2–2.1)[b] | 10.1 | 16 | 16.0 (9.2–26.0)[c] | 8.7 | 17 | 16.8 (9.8–26.9)[c] | 9.2 | 113 | 2.7 (2.3–3.3)[c] | 41.4 |

[a]$P<0.05$; [b]$P<0.01$; [c]$P<0.001$. [d]Difference between male and female relative risks significant at $P<0.05$. [e]Absolute excess risk per 10,000 person-years.

**Table III** Risks of second primary cancer by age at first treatment

| Age (years) | Lung cancer No. | Lung cancer RR (95% CI) | Lung cancer AR[d] | All other solid tumours No. | All other solid tumours RR (95% CI) | All other solid tumours AR[d] | Leukaemia No. | Leukaemia RR (95% CI) | Leukaemia AR[d] | Non-Hodgkin's lymphoma No. | Non-Hodgkin's lymphoma RR (95% CI) | Non-Hodgkin's lymphoma AR[d] | All malignancies except Hodgkin's No. | All malignancies except Hodgkin's RR (95% CI) | All malignancies except Hodgkin's AR[d] |
|---|---|---|---|---|---|---|---|---|---|---|---|---|---|---|---|
| <25 ($n$ = 890) | 0 | – | −0.1 | 5 | 3.6 (1.2–8.3)[a] | 5.9 | 5 | 42.0 (13.6–98.1)[c] | 8.1 | 0 | – | −0.2 | 10 | 6.0 (2.9–11.1)[c] | 13.8 |
| 25–44 ($n$ = 1179) | 6 | 7.1 (2.6–15.3)[c] | 6.8 | 17 | 2.4 (1.4–3.9)[b] | 13.2 | 8 | 32.1 (13.9–63.3)[c] | 10.2 | 5 | 15.3 (5.0–35.7)[c] | 6.2 | 36 | 4.3 (3.0–5.9)[c] | 36.3 |
| 45–54 ($n$ = 336) | 10 | 4.5 (2.2–8.4)[c] | 40.2 | 11 | 1.6 (0.8–2.8) | 20.9 | 2 | 10.3 (1.2–37.0)[a] | 9.3 | 3 | 13.8 (2.9–40.4)[b] | 14.4 | 26 | 2.7 (1.8–3.9)[c] | 84.3 |
| ⩾55 ($n$ = 441) | 16 | 3.0 (1.7–4.9)[c] | 61.3 | 15 | 1.0 (0.6–1.6) | −0.9 | 1 | 2.3 (0.1–12.8) | 3.2 | 9 | 24.5 (11.2–46.5)[c] | 49.5 | 41 | 1.9 (1.4–2.6)[c] | 111.8 |
| $\chi^2_1$ trend for relative risks | | 2.99 | | | 9.93[b] | | | 14.33[c] | | | 2.52 | | | 18.48[c] | |
| $\chi^2_1$ trend for absolute excess risks | | 22.90[c] | | | 0.01 | | | 0.13 | | | 27.57[c] | | | 17.74[c] | |

[a]$P<0.05$; [b]$P<0.01$; [c]$P<0.001$. [d]Absolute excess risk per 10,000 person-years. The absolute excess risks for all malignancies are in certain instances slightly greater than the sum of the four site-specific columns because they include myeloma and certain other lymphatic and haematopoietic tumours not included in any of these columns.

age-trends described above were restricted to particular treatment types. Adjustment of the relative risks in Table III for duration of follow-up, number of courses of treatment, and type of treatment, only marginally affected the results: the significant trends with age diminished slightly in magnitude, but remained highly significant.

Absolute excess risks of second cancer overall, of lung cancer, and of non-Hodgkin's lymphoma, increased greatly with age ($P<0.001$) (Table III), whereas for leukaemia and non-lung solid cancers there was no consistent relation of absolute excess risk to age.

We repeated the analyses of leukaemia risk by age, restricting the category under analysis to acute or non-lymphocytic leukaemias (ANLL) (15 cases). The ANLL analyses were imperfect, and therefore not used for the main analyses, because histological type and acuteness/chronicity were not stated for 15% of the leukaemias in the national cancer registration data set used as the comparison, but were known for all of the leukaemias in BNLI study patients. We also conducted the analyses for ANLL restricted to patients who had received any chemotherapy. In both instances the analyses showed a similar pattern to that seen in Table III for leukaemia overall: relative risks decreased highly significantly, by slightly more than tenfold, from age under 25 years to age 55 years and over, and absolute risks were about 2-fold lower for the age-groups over 45 years than for the age-groups younger than this.

Relative risks of second primary cancer overall varied significantly according to the histological subtype of the initial Hodgkin's disease (Table IV), although the range of risks was modest. Risks of non-Hodgkin's lymphoma (NHL) showed a much larger, significant variation with histology of the original Hodgkin's disease; greatest risk was after lymphocyte predominant Hodgkin's disease (RR = 55.6), and least with any cases after nodular sclerosing I (RR = 9.8), with no cases of NHL occurring after the few cases of lymphocyte depleted Hodgkin's disease. Risks of leukaemia and of non-lung solid malignancies were also significantly related to the histology of the initial Hodgkin's disease. When the relationship was re-tested omitting the cells in the table with no cases, the relationship for NHL remained highly significant ($P<0.001$) while those for leukaemia and for non-lung solid malignancies ceased to be significant. Adjustment for duration since first treatment, type of treatment and number of courses of treatment had only a slight and inconsistent effect on the results.

The relative risk of second primary cancer did not relate to the stage of Hodgkin's disease (Table V). This conclusion remained when the relationship was re-tested omitting the cells in the table (for leukaemia) with no cases, and was also not affected by adjustment of the risks for duration of follow-up, number of courses of treatment and type of treatment. Adjusting analyses by treatment type (Swerdlow *et al.*, 1992) for histology and stage had no consistent effect, and in no instance where convergence was possible did addition of these variables have a significant effect.

Risk of second cancers was also not significantly related to whether or not the patient had undergone splenectomy (Table VI). The relative risk of leukaemia was somewhat, but not significantly, greater in patients who had undergone splenectomy (RR = 19.3) than in those who had not (RR = 13.7). Adjustment for time since first treatment, type of treatment, and number of courses of treatment, marginally decreased each of the relative risks of second cancer in splenectomised compared to non-splenectomised patients, and the differences between the two groups remained non-significant.

### Discussion

There is now a great deal of evidence that modern intensive treatment of Hodgkin's disease causes leukaemia and solid second malignancies (Kaldor *et al.*, 1987; Tucker *et al.*,

**Table IV** Relative risks of second primary cancer by histological type of Hodgkin's disease

| | Lung cancer | | All other solid tumours | | Leukaemia | | Non-Hodgkin's lymphoma | | All malignancies except Hodgkin's | |
|---|---|---|---|---|---|---|---|---|---|---|
| *Histology* | *No.* | *RR (95% CI)* | *No.* | *RR (95% CI)* | *No.* | *RR (95% CI)* | *No.* | *RR (95% CI)* | *No.* | *RR (95% CI)* |
| Lymphocyte depleted | | 8.1 | | – | | – | | – | | 1.5 |
| ($n$ = 40) | 1 | (0.2–45.3) | 0 | | 0 | | 0 | | 1 | (0.0–8.6) |
| Lymphocyte predominant | | – | | 0.4 | | – | | 55.6 | | 1.7 |
| ($n$ = 168) | 0 | | 1 | (0.0–2.2) | 0 | | 5 | (18.0–129.7)$^c$ | 6 | (0.6–3.6) |
| Mixed cellularity | | 2.2 | | 1.4 | | 17.6 | | 22.1 | | 2.5 |
| ($n$ = 533) | 6 | (1.0–6.0)$^a$ | 10 | (0.7–2.6) | 4 | (4.8–45.1)$^c$ | 5 | (7.2–51.6)$^c$ | 25 | (1.6–3.7)$^c$ |
| Nodular sclerosing I | | 4.0 | | 1.8 | | 18.1 | | 9.8 | | 2.8 |
| ($n$ = 1317) | 16 | (2.3–6.5)$^c$ | 27 | (1.2–2.6)$^b$ | 9 | (8.3–34.3)$^c$ | 5 | (3.2–23.0)$^c$ | 57 | (2.2–3.6)$^c$ |
| Nodular sclerosing II | | 7.3 | | 1.4 | | 18.4 | | 11.9 | | 3.2 |
| ($n$ = 746) | 9 | (3.4–13.9)$^c$ | 7 | (0.6–2.9) | 3 | (3.8–53.8)$^c$ | 2 | (1.4–43.0)$^a$ | 21 | (2.0–4.8)$^c$ |
| All histologies$^d$ | | 3.8 | | 1.5 | | 16.2 | | 16.9 | | 2.7 |
| ($n$ = 2846) | 32 | (2.6–5.4)$^c$ | 45 | (1.1–2.0)$^a$ | 16 | 9.2–26.2)$^c$ | 17 | (9.9–27.1)$^c$ | 110 | (2.2–3.2)$^c$ |
| $\chi^2_4$ heterogeneity | 8.44 | | 12.19$^a$ | | 14.05$^b$ | | 24.25$^c$ | | 26.68$^c$ | |

$^a P<0.05$; $^b P<0.01$; $^c P<0.001$. $^d$Including 42 cases of unknown histology.

**Table V** Relative risks of second primary cancer by stage of Hodgkin's disease

| | Lung cancer | | All other solid tumours | | Leukaemia | | Non-Hodgkin's lymphoma | | All malignancies except Hodgkin's | |
|---|---|---|---|---|---|---|---|---|---|---|
| *Stage* | *No.* | *RR (95% CI)* | *No.* | *RR (95% CI)* | *No.* | *RR (95% CI)* | *No.* | *RR (95% CI)* | *No.* | *RR (95% CI)* |
| I ($n$ = 652) | 13 | 4.6 | 12 | 1.3 | 0 | – | 4 | 13.3 | 29 | 2.3 |
| | | (2.4–7.9)$^c$ | | (0.7–2.3) | | | | (3.6–34.0)$^c$ | | (1.5–3.2)$^c$ |
| II ($n$ = 847) | 10 | 5.1 | 16 | 2.0 | 5 | 19.2 | 5 | 18.6 | 36 | 3.4 |
| | | (2.5–9.4)$^c$ | | (1.2–3.3)$^a$ | | (6.2–44.7)$^c$ | | (6.0–43.4)$^c$ | | (2.4–4.8)$^c$ |
| III ($n$ = 809) | 5 | 2.3 | 14 | 1.7 | 8 | 29.2 | 5 | 18.1 | 32 | 2.9 |
| | | (0.7–5.3) | | (0.9–2.9) | | (12.6–57.5)$^c$ | | (5.9–42.1)$^c$ | | (2.0–4.1)$^c$ |
| IV ($n$ = 536) | 4 | 2.8 | 6 | 1.2 | 3 | 18.2 | 3 | 18.4 | 16 | 2.3 |
| | | (0.8–7.1) | | (0.4–2.5) | | (3.7–53.1)$^b$ | | (3.8–53.8)$^b$ | | (1.3–3.7)$^b$ |
| All stages$^d$ ($n$ = 2846) | 32 | 3.8 | 48 | 1.6 | 16 | 16.0 | 17 | 16.8 | 113 | 2.7 |
| | | (2.6–5.4)$^c$ | | (1.2–2.1)$^b$ | | (9.2–26.0)$^c$ | | (9.8–26.9)$^c$ | | (2.3–3.3)$^c$ |
| $\chi^2_1$ trend | 1.9 | | 0.004 | | 0.002 | | 0.19 | | 0.02 | |

$^a P<0.05$; $^b P<0.01$; $^c P<0.001$. $^d$Including 2 cases of unknown stage.

**Table VI** Relative risks of second primary cancer in relation to splenectomy

| | *Lung cancer* | | *All other solid tumours* | | *Leukaemia* | | *Non-Hodgkin's lymphoma* | | *All malignancies except Hodgkin's* | |
|---|---|---|---|---|---|---|---|---|---|---|
| | *No.* | *RR (95% CI)* | *No.* | *RR (95% CI)* | *No.* | *RR (95% CI)* | *No.* | *RR (95% CI)* | *No.* | *RR (95% CI)* |
| Splenectomy ($n$ = 1110) | 11 | 3.0 (1.8–6.6)[c] | 19 | 1.6 (1.0–2.5) | 8 | 19.3 (8.3–38.1)[c] | 8 | 18.0 (7.8–35.5)[c] | 46 | 2.9 (2.1–3.9)[c] |
| No splenectomy ($n$ = 1736) | 21 | 3.9 (2.4–5.9)[c] | 29 | 1.6 (1.0–2.2)[a] | 8 | 13.7 (5.9–26.9)[c] | 9 | 15.9 (7.3–30.1)[c] | 67 | 2.6 (2.1–3.4)[c] |

[a]$P<0.05$; [b]$P<0.01$; [c]$P<0.001$. In no instance is the difference between the splenectomy and no splenectomy risks statistically significant.

1988), and increasing data on the relation of these risks to specific treatments (Pedersen-Bjergaard *et al.*, 1987; Tucker *et al.*, 1988; Boivin & O'Brien, 1988; Kaldor *et al.*, 1990; Swerdlow *et al.*, 1992). There is considerable uncertainty, however, on the possible modifying effects of age and sex, and the histology and stage of Hodgkin's disease, on these risks. Such analyses need large data sets, but are of importance because they might indicate that different weight should be given to the risk of second primary cancers when selecting treatment for different categories of patient.

We found a greater relative risk of leukaemia (compared to general population expectations) for women than for men, but sex was not otherwise related to the relative risk of second primary cancers. Previous data on the sex ratio of second malignancies after Hodgkin's disease (Van der Velden *et al.*, 1988; Henry-Amar *et al.*, 1990) and after other radiotherapy (National Research Council, 1990) have been inconsistent.

We found relative risks of second cancers overall greater at younger ages than at older. This effect was most striking for leukaemia but significant also for solid cancers. For solid cancers, the absolute risk generally increased with age, whereas for leukaemia it did not. Therefore for leukaemia but not solid cancers, a possible interpretation is that an approximately constant risk with age on an additive scale may occur, which appears greater at younger than older ages when considered on a relative scale, because baseline (general population) rates are greater at older ages. Most previous studies have only analysed age-specific risks by comparison between groups *within* the study, without allowance for the difference in risk by age to be expected from general population rates. Results of such analyses for leukaemia have been inconsistent (Tester *et al.*, 1984; Valagussa *et al.*, 1986; Colman *et al.*, 1988; Tucker *et al.*, 1988; Van Leeuwen *et al.*, 1989; Henry-Amar *et al.*, 1990). Analyses of leukaemia relative risks by age compared to general population expectations, by van der Velden *et al.* (1988) and Tucker *et al.* (1988), found no relation to age. The difference from our results appears mainly to arise from a lower relative risk at older ages in our data than theirs. This does not appear to be due to less aggressive treatment at older ages in BNLI patients, since the age-gradient remained unchanged when we adjusted for treatment parameters. Nor was it due to the category of leukaemias analysed, since, despite methodological difficulties, it was clearly present separately for ANLL, which appears to be the type of leukaemia related to treatment (Kaldor *et al.*, 1990).

There do not appear to be previous data on solid cancer relative risks after Hodgkin's disease by age, although Tucker *et al.* (1988) commented without analysis on the young age at treatment of several of their patients with solid second primary cancers. The age relationship for solid cancers is likely to be of great practical importance. Unlike leukaemias, relative risks of solid tumours continue to be sizeably raised (and indeed may continue to increase) at least 10–20 years, and probably longer, after first treatment (Kaldor *et al.*, 1987; Tucker *et al.*, 1988; Henry-Amar *et al.*, 1990; Swerdlow *et al.*, 1992). Thus although absolute risks of solid tumours are fairly low in young patients, a continuation of (or increase in) high relative risks in these patients as they grow older, would translate into a very sizeable absolute risk of malignancy as background expected rates rise with age. This emphasises the need for further investigation of long-term risks, and for continued clinical follow-up of Hodgkin's disease patients and search for new less-carcinogenic treatment regimes.

We found no relationship of risk of leukaemia or solid second primary cancers to the histology of the initial Hodgkin's disease. Other authors too have found no significant relation for second leukaemias (Pedersen-Bjergaard *et al.*, 1987; van der Velden *et al.*, 1988; Kaldor *et al.*, 1990; Henry-Amar *et al.*, 1990). Risk of NHL, however, was significantly related to the histology of the original Hodgkin's disease, with greatest risk after lymphocyte predominant Hodgkin's disease. Clinical reports previously have noted an apparently much raised frequency of NHL after lymphocyte predominant tumours (Miettinen *et al.*, 1983; Bennett *et al.*, 1991). The only previous analysis of the risk, to our knowledge, also found greatest NHL risk after lymphocyte predominant Hodgkin's disease, although without histology review (Henry-Amar *et al.*, 1990). Review of the histology of the Hodgkin's disease and NHL tumours was carried out for the present study (by Dr M.H. Bennett and K. MacL.), and is of importance because a possible artefactual reason for an apparent relationship of NHL risk to particular Hodgkin's disease histologies would be that the initial tumours might have been composite ones, containing both Hodgkin's disease and NHL tissue. On review this was not the case for any of the cases reported here (Bennett *et al.*, 1991). Second primary NHL does not appear to relate to the type of treatment of the Hodgkin's tumour (Swerdlow *et al.*, 1992), but it is possible that part of the natural history of lymphocyte predominant Hodgkin's disease is a tendency to progress to NHL (Sundeen *et al.*, 1988; Hansmann *et al.*, 1989). Alternatively, immunological or other features of lymphocyte predominant Hodgkin's may predispose to NHL.

Risk of second primary cancer was unrelated to the stage of Hodgkin's disease at presentation. This was also found in previous studies for second primary leukaemia (Pedersen-Bjergaard *et al.*, 1987; Van der Velden *et al.*, 1988; Colman *et al.*, 1988) and solid cancers (Henry-Amar, 1988), at least after adjusting for variation in treatment by stage. Kaldor *et al.* (1990), in a case-control study, found leukaemia risk lower after stage I than after later stages of Hodgkin's disease. No leukaemia cases occurred after stage I Hodgkin's disease in our data, which was in the direction of Kaldor's findings, although the relationship of stage to risk was not significant.

Certain recent reports have found a significant raised risk of leukaemia in Hodgkin's disease patients who have undergone splenectomy compared to those who have not (Van der Velden *et al.*, 1988; Van Leeuwen *et al.*, 1989; Kaldor *et al.*, 1990), and have suggested that the benefit of splenectomy needs to be balanced against this potential disbenefit (Van Leeuwen *et al.*, 1989). We, like some other investigators (Tucker *et al.*, 1988; Henry-Amar *et al.*, 1990), found a smaller non-significant increase in leukaemia risk after splenectomy. Adjustment for potentially confounding variables did not appreciably alter this. There was no suggestion of raised risk of other malignancies after splenectomy in a previous analysis (Henry-Amar *et al.*, 1990) or in our data.

We thank the collaborators in the BNLI whose patients are included in these analyses, and the regional cancer registries in England and Wales who assisted in follow-up of patients. We pay tribute to Dr M.H. Bennett who examined the pathology of many of the BNLI cases and was a major contributor to the work of the BNLI over many years, and who died recently. We are grateful to Miss J. Bonner and Mrs E. Middleton for secretarial help. The Epidemiological Monitoring Unit is funded by the Medical Research Council. The BNLI thanks the Lymphoma Research Trust, the Cancer Research Campaign, the Lisa Lear Fund and the Isle of Man Anti-Cancer Association for funding.

### References

BENNETT, M.H., MACLENNAN, K.A., EASTERLING, M.J., VAUGHAN HUDSON, B., VAUGHAN HUDSON, G. & JELLIFFE, A.M. (1985). Analysis of histological subtypes in Hodgkin's disease in relation to prognosis and survival. In *The Cytobiology of Leukaemias and Lymphomas*. Quaglino, D. & Hayhoe, F.G.J. (eds). Serono Symposia, Vol. 20, pp. 15–32. Raven Press: New York.

BENNETT, M.H., MACLENNAN, K.A. VAUGHAN HUDSON, G. & VAUGHAN HUDSON, B. (1991). Non-Hodgkin's lymphoma arising in patients treated for Hodgkin's disease in the BNLI: a 20 year experience. *Ann. Oncol.*, **2** (Suppl 2), 83–92.

BOIVIN, J.-F., HUTCHISON, G.B., LYDEN, M., GODBOLD, J., CHOROSH, J. & SCHOTTENFELD, D. (1984). Second primary cancers following treatment of Hodgkin's disease. *J. Natl Cancer Inst.*, **72**, 233–241.

BOIVIN, J.-F. & O'BRIEN, K. (1988). Solid cancer risk after treatment of Hodgkin's disease. *Cancer*, **61**, 2541–2546.

BRESLOW, N.E. & DAY, N.E. (1987). Statistical Methods in Cancer Research Volume II – *The Design and Analysis of Cohort Studies*. IARC Scientific Publication, No. 82. International Agency for Research on Cancer: Lyon.

CARBONE, P.P., KAPLAN, H.S., MUSSHOFF, K., SMITHERS, D.W. & TUBIANA, M. (1971). Report of the committee on Hodgkin's disease staging. *Cancer Res.*, **31**, 1860–1861.

COLEMAN, M., DOUGLAS, A. & HERMON, C. (1986). Cohort study analyses with a FORTRAN computer program. *Int. J. Epidemiol.*, **15**, 134–137.

COLMAN, M., EASTON, D.F., HORWICH, A. & PECKHAM, M.J. (1988). Second malignancies and Hodgkin's disease – The Royal Marsden Hospital experience. *Radiother. Oncol.*, **11**, 229–238.

HANSMANN, M.-L., STEIN, H., FELLBAUM, C., HUI, P.K., PARWARESCH, M.R. & LENNERT, K. (1989). Nodular paragranuloma can transform into high-grade malignant lymphoma of B type. *Hum. Pathol.*, **20**, 1169–1175.

HENRY-AMAR, M. (1988). Quantitative risk of second cancer in patients in first complete remission from early stages of Hodgkin's disease. *NCI Monogr.*, **6**, 65–72.

HENRY-AMAR, M., AEPPLI, D.M., ANDERSON, J., ASHLEY, S., BONICHON, F., COX, R.S., DAHLBERG, S.J., DEBOER, G., DIXON, D.O., GOBBI, P.G., GREGORY, W., HASENCLEVER, D., LÖFFLER, M., POMPE KIRN, V., SANTARELLI, M.T., SPECHT, L., SWINDELL, R. & VAUGHAN HUDSON, B. (1990). Study of second cancer risk. In *Treatment Strategy in Hodgkin's Disease*. Somers, R., Henry-Amar, M., Meerwaldt, J.H. & Carde, P. (eds), pp. 355–380. Colloques INSERM/John Libbey Eurotext: Paris.

KALDOR, J.M., DAY, N.E., BAND, P., CHOI, N.W., CLARKE, E.A., COLEMAN, M.P., HAKAMA, M., KOCH, M., LANGMARK, F., NEAL, F.E., PETTERSSON, F., POMPE-KIRN, V., PRIOR, P. & STORM, H.H. (1987). Second malignancies following testicular cancer, ovarian cancer and Hodgkin's disease: an international collaborative study among cancer registries. *Int. J. Cancer*, **39**, 571–585.

KALDOR, J.M., DAY, N.E., CLARKE, E.A., VAN LEEUWEN, F.E., HENRY-AMAR, M., FIORENTINO, M.V. & 22 others (1990). Leukemia following Hodgkin's disease. *New Engl. J. Med.*, **322**, 7–13.

MIETTINEN, M., FRANSSILA, K.O. & SAXÉN, E. (1983). Hodgkin's disease, lymphocyte predominance nodular. Increased risk for subsequent non-Hodgkin's lymphomas. *Cancer*, **51**, 2293–2300.

NATIONAL RESEARCH COUNCIL (1990). *Health Effects of Exposure to Low Levels of Ionizing Radiation*. Bier, V. National Academy Press: Washington DC.

PEDERSEN-BJERGAARD, J., SPECHT, L., LARSEN, S.O., ERSBØLL, J., STRUCK, J., HANSEN, M.M., HANSEN, H.H. & NISSEN, N.I. (1987). Risk of therapy-related leukaemia and pre-leukaemia after Hodgkin's disease. Relation to age, cumulative dose of alkylating agents, and time from chemotherapy. *Lancet*, **ii**, 83–88.

STATISTICS AND EPIDEMIOLOGY RESEARCH CORPORATION (1989). EGRET. *Statistics and Epidemiology Research Corporation*: Seattle.

SUNDEEN, J.T., COSSMAN, J. & JAFFE, E.S. (1988). Lymphocyte predominant Hodgkin's disease nodular subtype with coexistent 'large cell lymphoma'. Histological progression or composite malignancy? *Am. J. Surg. Pathol.*, **12**, 599–606.

SWERDLOW, A.J., DOUGLAS, A.J., VAUGHAN HUDSON, G., VAUGHAN HUDSON, B., BENNETT, M.H. & MACLENNAN, K.A. (1992). Risk of second primary cancers after Hodgkin's disease by type of treatment: analysis of 2846 patients in the British National Lymphoma Investigation. *Br. Med. J.*, **304**, 1137–1143.

TESTER, W.J., KINSELLA, T.J., WALLER, B., MAKUCH, R.W., KELLEY, P.A., GLATSTEIN, E. & DEVITA, V.T. (1984). Second malignant neoplasms complicating Hodgkin's disease: the National Cancer Institute experience. *J. Clin. Oncol.*, **2**, 762–769.

TUCKER, M.A., COLEMAN, C.N., COX, R.S., VARGHESE, A. & ROSENBERG, S.A. (1988). Risk of second cancers after treatment for Hodgkin's disease. *New Engl. J. Med.*, **318**, 76–81.

VALAGUSSA, P., SANTORO, A., FOSSATI-BELLANI, F., BANFI, A. & BONADONNA, G. (1986). Second acute leukemia and other malignancies following treatment for Hodgkin's disease. *J. Clin. Oncol.*, **4**, 830–837.

VAN DER VELDEN, J.W., VAN PUTTEN, W.L.J., GUINEE, V.F., PFEIFFER, R., VAN LEEUWEN, F.E., VAN DER LINDEN, E.A.M., VARDOMSKAYA, I., LANE, W., DURAND, M., LAGARDE, C., HAGEMEISTER, F.B., HAGENBEEK, A. & EGHBALI, H. (1988). Subsequent development of acute non-lymphocytic leukemia in patients treated for Hodgkin's disease. *Int. J. Cancer*, **42**, 252–255.

VAN LEEUWEN, F.E., SOMERS, R., TAAL, B.G., VAN HEERDE, P., COSTER, B., DOZEMAN, T., HUISMAN, S.J. & HART, A.A.M. (1989). Increased risk of lung cancer, non-Hodgkin's lymphoma, and leukemia following Hodgkin's disease. *J. Clin. Oncol.*, **7**, 1046–1058.

WORLD HEALTH ORGANIZATION (1967). *Manual of the International Statistical Classification of Disease, Injuries, and Causes of Death*. Eighth Revision. WHO: Geneva.

WORLD HEALTH ORGANIZATION (1977). *Manual of the International Statistical Classification of Diseases, Injuries, and Causes of Death*. Ninth Revision. WHO: Geneva.